# A case of surgical treatment for recurrence of right ventricular metastasis due to renal cell carcinoma after molecular targeted therapy

**DOI:** 10.1186/s40792-024-01940-8

**Published:** 2024-06-04

**Authors:** Keita Sasaki, Naritomo Nishioka, Mika Yamamoto, Kenichi Kato, Ryo Matsumoto, Takahiko Masuda, Ryushi Maruyama, Yoshihiko Kurimoto, Akira Yamada, Shuichi Naraoka

**Affiliations:** Department of Cardiovascular Surgery, Teine Keijinkai Hospital, 1-12-1-40 Maeda, Teine-Ku, Sapporo, 006-8555 Japan

**Keywords:** Cardiac tumor, Cardiac metastases, Renal cell carcinoma, Molecularly targeted therapies

## Abstract

**Background:**

Cardiac metastasis including the right ventricle from renal cell carcinoma is rare. No standard treatment for cardiac metastasis and recurrence in renal cell carcinoma has been established.

**Case presentation:**

We present the case of a 61-year-old man who underwent the resection of recurrent right ventricular metastasis caused by renal cell carcinoma following molecular targeted therapy. The first cardiac operation was performed for right ventricular metastasis due to renal cell carcinoma. The patient had a good postoperative course. Two years after the first operation, however, follow-up computed tomography revealed the recurrence of the right ventricular tumor and metastases in both lungs. Molecular targeted therapy was carried out and effectively controlled the lung metastasis but the right ventricular lesion remained unchanged, leading to reoperation. The recurrent right ventricular tumor was completely resected through a redo median sternotomy assisted by cardiopulmonary bypass. The patient had an uneventful postoperative course and was discharged on the 13th postoperative day. Follow-ups at 2 years showed no cardiac recurrence.

**Conclusion:**

Surgical intervention was considered useful in managing the recurrence of right ventricular metastasis from renal cell carcinoma after molecular targeted therapy.

## Background

Renal cell carcinoma (RCC) is known for its tendency to metastasize hematogenously, primarily due to the abundant blood flow of the kidneys [1]. The common metastatic sites of RCC include the lungs, liver, bones, soft tissues, and central nervous system [2]. Cardiac metastasis including the right ventricle is rare. We previously reported a case involving the surgical treatment of isolated right ventricular (RV) metastasis from RCC [3]. Unfortunately, this case experienced a recurrence of RV metastasis 2 years after the first operation during subsequent follow-ups. Here, we successfully managed the recurrent RV metastasis through surgical intervention following molecular targeted therapy, demonstrating the utility of this approach in the management of such cases.

## Case presentation

A 61-year-old man presented to our hospital with progressive dyspnea and medical history of RCC. At the age of 51, he underwent partial nephrectomy for RCC, classified as histopathological stage I. Two years after the initial operation, multiple bilateral lung metastases were identified. The patient was treated with molecular targeted therapy (sorafenib, 400 mg/day), which led to the disappearance of these metastatic lesions. However, at the age of 58, a metastasis in the right adrenal gland was detected. A right adrenalectomy was performed following shrinkage of the metastatic lesion by alternative molecular targeted therapy (sunitinib, 50 mg/day).

**Fig. 1:**
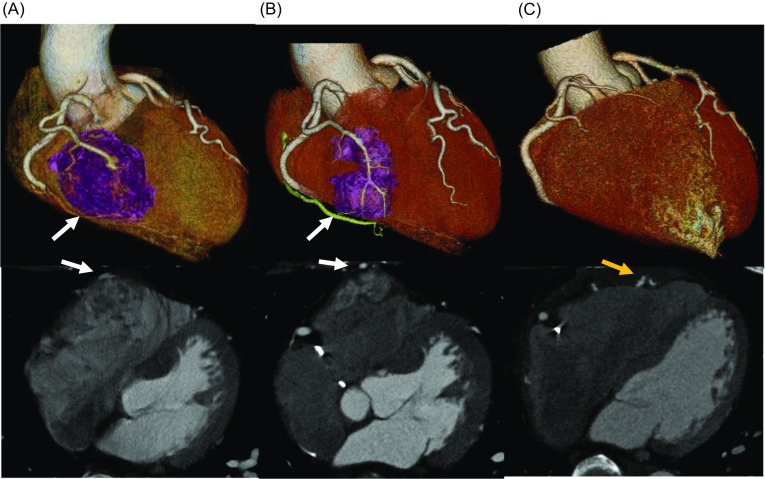
Enhanced three-dimensional computed tomography showing the RV metastasis due to renal cell carcinoma before the first operation (**A**), the recurrence of the RV (**B**) (each purple area), and the complete removal of the RV tumor after reoperation (**C**). White arrows show the RV branch of the right coronary artery. A yellow arrow shows the Hemashield Woven Double Velour Cardiovascular Fabric^®^. *RV* right ventricular

A concomitant computed tomography (CT) scan revealed a tumor on the free wall of the RV, not involving the inferior vena cava (IVC) (Fig. 1A). To address this RV tumor, resection was undertaken with cardiopulmonary bypass, carefully avoiding transmural wall resection to prevent postoperative RV dysfunction. In other words, the RV tumor was removed by scraping. Cryoablation was applied to the cut surface of the RV wall to mitigate recurrence and ventricular arrhythmia risks. The patient was discharged on the 16th postoperative day without major complications. Histopathological examination confirmed the RV tumor as a metastasis originating from RCC. From the condition of the specimens, it was impossible to determine whether the resection edges were positive or not. An oncologist managed the patient’s postoperative follow-up; this case has been previously reported by us [3].

**Fig. 2:**
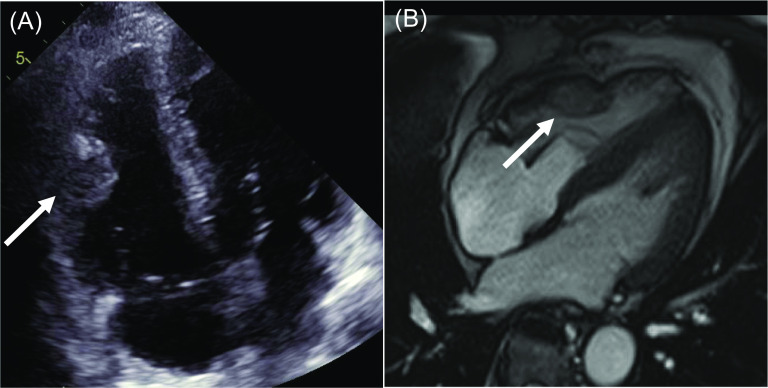
Each white arrow indicates the RV tumor. **A** Four-chamber view on transthoracic echocardiography. **B** Cardiac magnetic resonance imaging shows the clear margins of the cardiac tumor. *RV* right ventricular

Two years after the initial resection of the RV tumor, the patient remained asymptomatic during clinical examinations. However, a CT scan revealed the recurrence of the RV tumor and bilateral lung tumors. The metastases of the bilateral lungs were once again under control following the administration of molecular targeted therapy (axitinib, 10 mg/day). The right ventricular lesion remained unchanged. Transthoracic echocardiography revealed a 36 mm × 36 mm mass on the free wall of the RV (Fig. 2A), and the patient’s cardiac function was preserved. Cardiac magnetic resonance imaging revealed a hypervascular mass on the free wall of the RV (Fig. 2B). Contrast-enhanced cardiac CT demonstrated the intramyocardial mass in the free wall of the RV, supplied by the RV branch of the right coronary artery (Fig. 1B). These findings were generally consistent with previous findings. Based on the overall assessment, this case was diagnosed as a local recurrence of metastasis to the RV from RCC. Given the potential impact of the RV lesion on the patient’s prognosis, after discussions with cardiac surgeons, urologists, the patient and the patient’s family, the decision was made to proceed with reoperation.

**Fig. 3:**
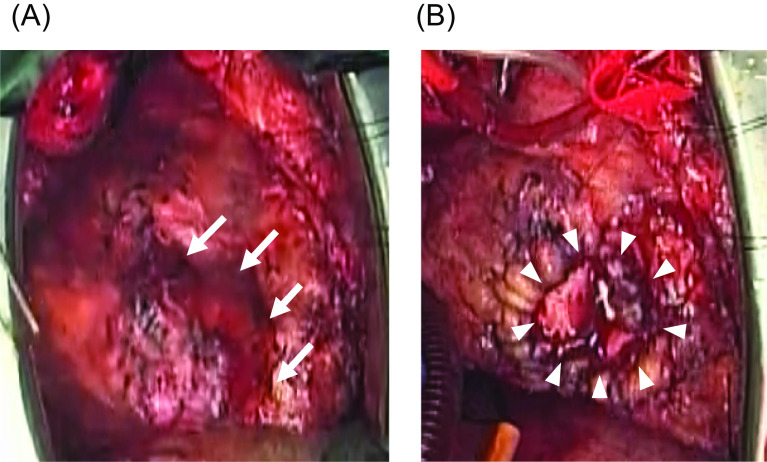
Intraoperative findings. **A** After re-sternotomy, the tumor was found on the RV surface (white arrows). **B** The resected RV wall was repaired with a patch graft (white triangles). *RV* right ventricular

Under general anesthesia and through a redo median sternotomy, a cardiopulmonary bypass was established between the ascending aorta and bicaval drainage. The tumor, observed on the free wall of the RV, measured 5 cm × 5 cm (Fig. 3A). After cardiac arrest induced by antegrade and retrograde cardioplegia, the right atrium was opened to assess the tricuspid valve, which was found to be intact. Subsequently, the tumor lesion of the RV wall was completely resected using a No.11 scalpel. Cryoablation was then performed on the cut surface of the RV wall to mitigate the risk of recurrence and ventricular arrhythmia. The resected RV wall was repaired with patch grafts (Hemashield Woven Double Velour Cardiovascular Fabric^®^, Intervascular SAS, La Ciotat, France) and 4-0 monofilament polypropylene sutures (Fig. 3B).

**Fig. 4:**
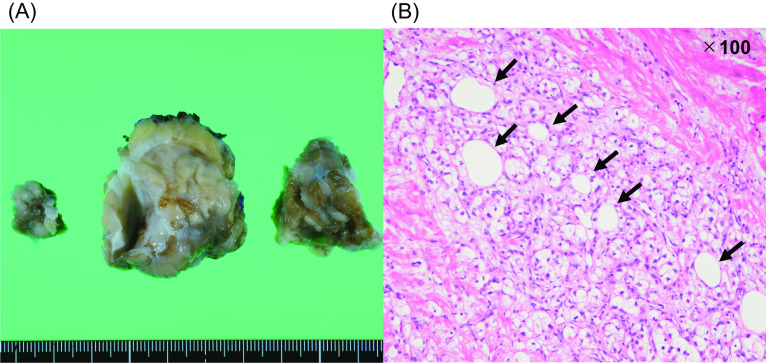
Macroscopic (**A**) and microscopic (**B**) findings for the RV tumor. Blank arrows indicate clear cytoplasm, which indicates metastasis due to renal cell carcinoma (hematoxylin and eosin stains). *RV* right ventricular

Postoperative contrast-enhanced cardiac CT revealed the complete removal of the RV tumor (Fig. 1C). The patient experienced an uneventful recovery and was discharged on the 13th postoperative day. The pathological examination of the RV tumor indicated clear cell carcinoma, confirming its metastatic origin from RCC. However, since the tumor could not be resected en bloc, it was impossible to perform the pathological evaluation as the tumor edge (Fig. 4). The patient remained recurrence-free in the two years after the reoperation.

## Discussion

Here, we presented the utility of surgical intervention in managing the recurrence of right ventricular metastasis due to renal cell carcinoma following molecular targeted therapy. Previous studies have reported that cardiac tumors predominantly arise from metastatic rather than primary causes. Nova-Camacho et al. reviewed 1294 autopsies involving adults diagnosed with various tumors, revealing that out of 133 cardiac tumors, only 9 were primary, while 124 were secondary tumors, emphasizing the prevalence of metastatic cardiac tumors [4].

The pathways for cardiac metastasis include direct extension, hematogenous spread, lymphatic dissemination, and transvenous extension [5]. The transvenous pathway is particularly associated with RCC, hepatocellular carcinoma, and leiomyoma [5]. Cardiac metastasis can affect various cardiac structures, including the pericardium, myocardium, epicardium, endocardium, and the right heart system. It is of note that there are few reports of metastatic tumors occurring in the right heart system that do not involve the IVC [5].

To date, no standard treatment for metastatic cardiac tumor has been established. The median survival for RCC with metastasis is 5 months, and the 1-year survival rate is 29% [6]. Several reports have demonstrated partial responses to systemic agents in treating cardiac metastasis from RCC [7–9]. In addition, one systematic review showed that complete resection of metastases from RCC was consistently beneficial in overall and cancer-specific survival [10]. Although not described for cardiac metastases, this review reported the 5-year survival rate of 37–54% for complete resection of lung metastases and 0–24% for failure to resect completely, indicating that complete resection of metastases might be beneficial. Another study reported the median overall survival period of 80.1 months for patients who underwent resection of metastases [11]. In another study, patients who underwent compete resection of metastases had the median cancer-specific survival period of 4.8 years and the 5-year cancer-specific survival rate of 49.4%, compared with 1.3 years and 19%, respectively, in the incomplete resection group [12]. These reports have suggested that resection of metastases from RCC prolonged patients’ prognosis. Based on these findings, the Japanese clinical practice guideline for renal cancer states that resection of metastases is expected to improve survival in patients who have good performance status, a long disease-free interval, and the possibility of complete resection [13].

On the other hand, the same literature recommends molecular targeted therapy for advanced renal cancer. The combination of molecular targeted therapy and resection of RV metastasis from RCC appears promising, as evidenced by the survival of our patient for 2 years post-initial cardiac surgery despite recurrence.

In this case, the recurrence of RCC metastasis in the right ventricle recurred at the same site 2 years after the initial resection. During the initial cardiac operation, we opted not to resect the transmural right ventricular wall to prevent postoperative right heart failure. The metastatic site may have remained within the right ventricular wall, eventually leading to the enlargement of a residual RV tumor. If the RV tumor had been removed transmurally during the initial operation, the outcome might have been different. Although the recurrence of RV metastasis was noted, this patient underwent reoperation and had a favorable outcome. This report suggests that surgical resection is an effective treatment for metastatic cardiac tumors.

Despite significant advancements in the treatment of malignant tumors, cardiac metastases, especially in the context of RCC, continue to pose challenges and are associated with a poor prognosis. Although the combination of surgical resection and molecular targeted therapy, as demonstrated in this case, may be effective against metastatic cardiac tumors, there is a need for additional cases to further substantiate treatment outcomes.

## Conclusions

We successfully treated the recurrent right ventricular metastasis of renal cell carcinoma with cardiac surgery following molecular targeted therapy. This case highlights that surgical resection remains a valuable strategy for managing the recurrence of cardiac tumors.

## Data Availability

There is no additional date to disclose.

## Abbreviations

CT: Computed tomography
IVC: Inferior vena cava
RCC: Renal cell carcinoma
RV: Right ventricular
